# Regression Analysis for Constraining Free Parameters in Electrophysiological Models of Cardiac Cells

**DOI:** 10.1371/journal.pcbi.1000914

**Published:** 2010-09-02

**Authors:** Amrita X. Sarkar, Eric A. Sobie

**Affiliations:** Department of Pharmacology and Systems Therapeutics, Mount Sinai School of Medicine, New York, New York, United States of America; University of California San Diego, United States of America

## Abstract

A major challenge in computational biology is constraining free parameters in mathematical models. Adjusting a parameter to make a given model output more realistic sometimes has unexpected and undesirable effects on other model behaviors. Here, we extend a regression-based method for parameter sensitivity analysis and show that a straightforward procedure can uniquely define most ionic conductances in a well-known model of the human ventricular myocyte. The model's parameter sensitivity was analyzed by randomizing ionic conductances, running repeated simulations to measure physiological outputs, then collecting the randomized parameters and simulation results as “input” and “output” matrices, respectively. Multivariable regression derived a matrix whose elements indicate how changes in conductances influence model outputs. We show here that if the number of linearly-independent outputs equals the number of inputs, the regression matrix can be inverted. This is significant, because it implies that the inverted matrix can specify the ionic conductances that are required to generate a particular combination of model outputs. Applying this idea to the myocyte model tested, we found that most ionic conductances could be specified with precision (R^2^ > 0.77 for 12 out of 16 parameters). We also applied this method to a test case of changes in electrophysiology caused by heart failure and found that changes in most parameters could be well predicted. We complemented our findings using a Bayesian approach to demonstrate that model parameters cannot be specified using limited outputs, but they can be successfully constrained if multiple outputs are considered. Our results place on a solid mathematical footing the intuition-based procedure simultaneously matching a model's output to several data sets. More generally, this method shows promise as a tool to define model parameters, in electrophysiology and in other biological fields.

## Author Summary

Mathematical models of biological processes generally contain many free parameters that are not known from experiments. Choosing values for these parameters, although an important step in the construction of realistic computational models, is frequently performed using an ad hoc approach that is a combination of intuition and trial and error. We have developed a novel method for constraining free parameters in mathematical models based on the techniques of linear algebra. We demonstrate this method's utility through simulations with a model of a human heart cell. The underlying premise is that if the model is only asked to recapitulate one or a few biological behaviors, the values of the parameters may be ambiguous; however, if the model must simultaneously match many features of experimental data, the free parameters can be determined uniquely. The results demonstrate that if computational models are to be realistic, they must be compared with several sets of data at the same time. This new method should serve as a valuable tool for investigators interested in developing realistic mathematical models of biological processes.

## Introduction

Mathematical modeling has become an increasingly popular and important technique for gaining insight into biological systems, both in physiology, where models have a long history [1], [2], and in biochemistry and cell biology, where quantitative approaches have gained traction more recently [3], [4]. However, as new models proliferate and become increasingly complex, analysis of parameter sensitivity has emerged as an important issue [5], [6]. It is clear that to understand a model requires not only knowing the output generated using the published “baseline” set of parameters, but also some knowledge of how changes in the model's parameters affect its behavior.

During the development of a mathematical model, the choice of parameters is a critical step. Parameters are constrained by data whenever this is possible, but direct measurements are frequently lacking. Often, however, a situation exists in which values for many parameters are unknown, but a considerable amount is known about the system's emergent phenomena. In such cases, experienced researchers narrow down the values of the unknown model parameters based on how the model “ought to behave.” Parameter sets that generate grossly unrealistic output are rejected whereas those that produce reasonable output are tentatively accepted until they fail in some important respect. The emergent phenomena considered in this process can be switching or oscillatory behavior in the case of biochemical signaling models [3], [4], or outputs such as action potential (AP) and calcium transient morphology in models of ion transport [7]–[10]. Computational studies, however, have revealed the limitations of this intuition-based procedure. In particular, work in theoretical neuroscience has shown that when a single output such as neuronal firing rate is considered, many different combinations of model parameters can generate equivalent behavior [11]–[14].

**Figure 1:**
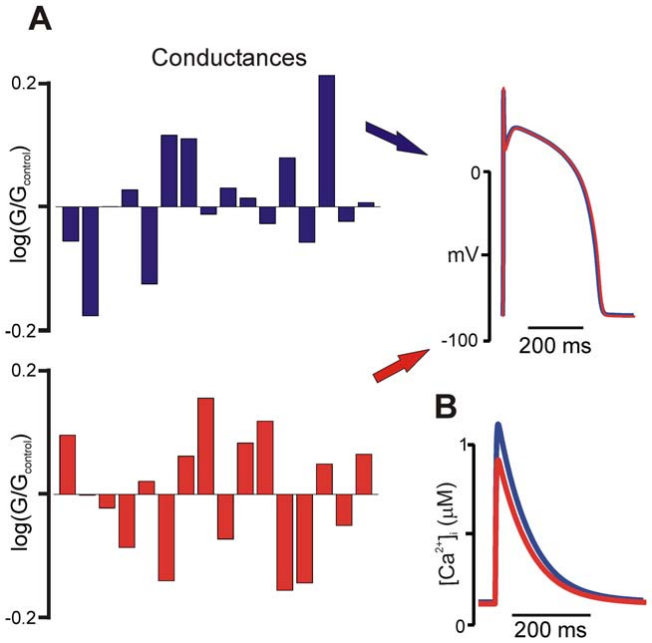
Effects of parameter variation on model output. (**A**) Drastically different combinations of ionic conductances result in nearly identical action potential morphology. The bar graphs show log(G/G_control_) for each ionic conductance in the TNNP [15] model, where G_control_ is the conductance in the published model (see Table 1 in Text S1 for full list) (**B**) Intracellular calcium (Ca^2+^) transients produced by the two parameter combinations are distinct, in terms of both amplitude and kinetics, suggesting that such information could be used to distinguish between the two parameter sets.

This general problem is illustrated in Figure 1A, which shows results from a popular mathematical model of the human ventricular action potential, that of ten Tusscher, Noble, Noble, and Panfilov (TNNP; [15]). Random variation of model parameters revealed that completely different parameter combinations could produce virtually identical AP morphology. This result is analogous to studies by Prinz et al. examining firing rate in neuronal cell models [13], [14]. However, an interesting aspect of the simulation is as follows. The two hypothetical cells, although generating nearly identical APs under normal conditions, exhibited intracellular Ca^2+^ transients that differed with respect to both amplitude and kinetics (Figure 1B). Theoretically, then, a justifiable choice between these two parameter combinations, while impossible based only on the results shown in Figure 1A, could be made by considering the additional information in Figure 1B. Such distinctions are frequently made by researchers with experimental expertise, who either accept or reject models based on how well they recapitulate a range of observed phenomena. This process, although somewhat arbitrary and potentially subject to bias, nonetheless reflects sound reasoning, since a “good” model should successfully reproduce many biological behaviors.

Based on results such as those shown in Figure 1, we sought to formalize and place on a sound mathematical footing the process of choosing parameters by comparing model output with several sets of data. In particular, our hypothesis was that examining a single model output, such as action potential duration (APD), would fail to constrain parameters, but success would be more likely if the number of physiological outputs was similar to the number of free model parameters. We demonstrate that this is true in the case of the TNNP model [15] through two methods. The first, an extension of the use of multivariable regression for parameter sensitivity analysis [16], consists of inverting a regression matrix and then using this to calculate the changes in model parameters required to generate a given change in outputs. The second method employs Bayes's theorem to estimate the probabilities that model parameters lie within certain ranges. The results, which are generally applicable across different models and different biological systems, can be of great use when building new models, and also provide new insights into the relationships between model parameters and model results.

## Results

The overall hypothesis of our study was that if several physiologically-relevant characteristics of a model's behavior were known, this information would be sufficient to constrain some or all of the model's parameters. We tested this idea using two approaches: one based on multivariable regression and the other based on Bayes's theorem. We began by generating a database of candidate models. The parameters that define maximal conductances and rates of ion transport in the TNNP model [15] were varied randomly, and several simulations, defining how the candidate model responded to altered experimental conditions, were performed with each new set of parameters. In general, the simulations reflected experimental tests commonly performed on ventricular myocytes, such as determining the threshold for excitation or changing the rate of pacing.

For the first approach, the results of these simulations were collected in “input” and “output” matrices **X** and **Y**, respectively. Each column of **X** corresponded to a model parameter, and each row corresponded to a candidate model (n = 300). The columns of **Y** were the physiological outputs extracted from the simulation results, such as action potential duration (APD) and Ca^2+^ transient amplitude. Complete descriptions of the randomization procedure and simulation protocols are provided in the Methods and Text S1. Outputs are listed in Table 1 and described in detail in Text S1.

**Table 1:** Physiological outputs in simulations with TNNP model.

Output #	Abbreviation	Description
**1**	**APD**	**Action potential duration**
**2**	**V_rest_**	**Resting membrane potential**
**3**	**V_peak_**	**Peak voltage during phase 0**
4	dV/dt _max_	Maximum upstroke velocity
**5**	**ΔCa**	**Ca^2+^ transient amplitude**
6	V_maxmin_	Shape parameter to characterize AP
7	V_minmax_	Shape parameter to characterize AP
8	t_minmax_	Shape parameter to characterize AP
9	BCL_alt_	Alternans threshold
**10**	**I_thresh_**	**Stimulation threshold**
11	Maxslope	Maximum slope of restitution curve
12	Time to peak	Time to peak of Ca^2+^ transient
**13**	**Decay time**	**Time constant of decay of Ca^2+^ transient**
14	APD_pause_	Action potential duration after a long pause
15	ΔCa_pause_	Ca^2+^ transient amplitude after a long pause
16	V_maxmin_pause_	Shape parameter to characterize AP after a long pause
17	V_minmax_pause_	Shape parameter to characterize AP after a long pause
18	t_minmax_pause_	Shape parameter to characterize AP after a long pause
**19**	**APD_diff_**	**Difference in APD between the first and the last AP during pacing**
**20**	**ΔCa_diff_**	**Difference in ΔCa between the first and the last AP during pacing**
**21**	**APD_hypo_**	**Action potential duration during hypokalemia**
**22**	**APD_hyper_**	**Action potential duration during hyperkalemia**
23	V_maxmin hypo_	Shape parameter to characterize AP during hypokalemia
**24**	**V_minmax hypo_**	**Shape parameter to characterize AP during hypokalemia**
25	t_minmax hypo_	Shape parameter to characterize AP during hypokalemia
**26**	**V_maxmin hyper_**	**Shape parameter to characterize AP during hyperkalemia**
**27**	**V_minmax hyper_**	**Shape parameter to characterize AP during hyperkalemia**
28	t_minmax hyper_	Shape parameter to characterize AP during hyperkalemia
**29**	**Ca^2+^**	**Intracellular Ca^2+^ after 60 seconds of quiescence**
**30**	**Na^+^**	**Intracellular Na^+^ after 60 seconds of quiescence**
**31**	**K^+^**	**Intracellular K^+^ after 60 seconds of quiescence**
**32**	**Frequency**	**Frequency adaptation of APD**

For each set of random parameters, simulations were performed to calculate each output in the Table. Methods used for calculation of these outputs are provided in Text S1. Entries in bold and plain text, respectively, indicate outputs that were retained or rejected for matrix inversion.

**Figure 2:**
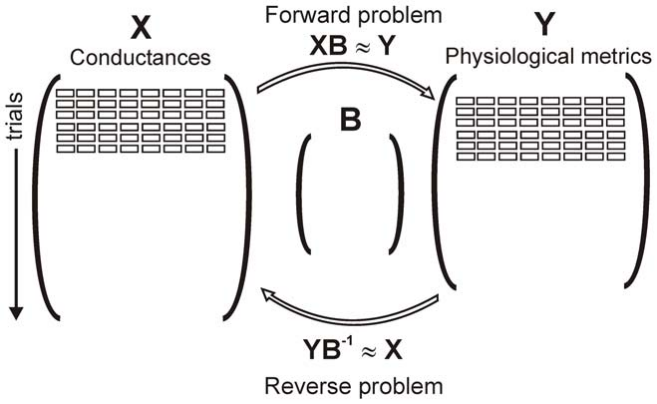
Schematic of input, output and regression matrix structures. Randomly-varied model parameters are collected in an input matrix **X** with dimensions *n*, corresponding to the number of trials, by *p*, corresponding to the number of parameters. Simulation results define *m* outputs that are collected in the output matrix **Y**, with dimensions an *n*×*m*. Regression matrix **B**, with dimensions *p*×*m*, can be used to predict **Y** from **X**, the so-called “forward problem.” If *m* = *n*, and the outputs are linearly independent, then **B** can be inverted, and **YB^−1^** should be a good approximation of **X**. This is our strategy for addressing the “reverse problem.”

Multivariable regression techniques were used to quantitatively relate the inputs to the outputs. In the “forward problem,” a matrix of regression coefficients **B** was derived such that the predicted output **Y** ^ = **XB** was a close approximation of the actual output **Y**. This method has recently been proven useful for characterizing the parameter sensitivity of electrophysiological models [16]. We reasoned that a similar approach could be used to address the question: if the measurable physiological characteristics of a cardiac myocyte are known, can this information be used to uniquely specify the magnitudes of the ionic currents and Ca^2+^ transport processes? Specifically, we hypothesized that if: 1) **Y** ^ = **XB** was a close approximation of the true output **Y**, and 2) **B** was a square matrix of full rank, then **X_predicted_** = **YB^−1^** should be a close approximation of the true input matrix **X**. This argument is illustrated schematically in Figure 2.

**Figure 3:**
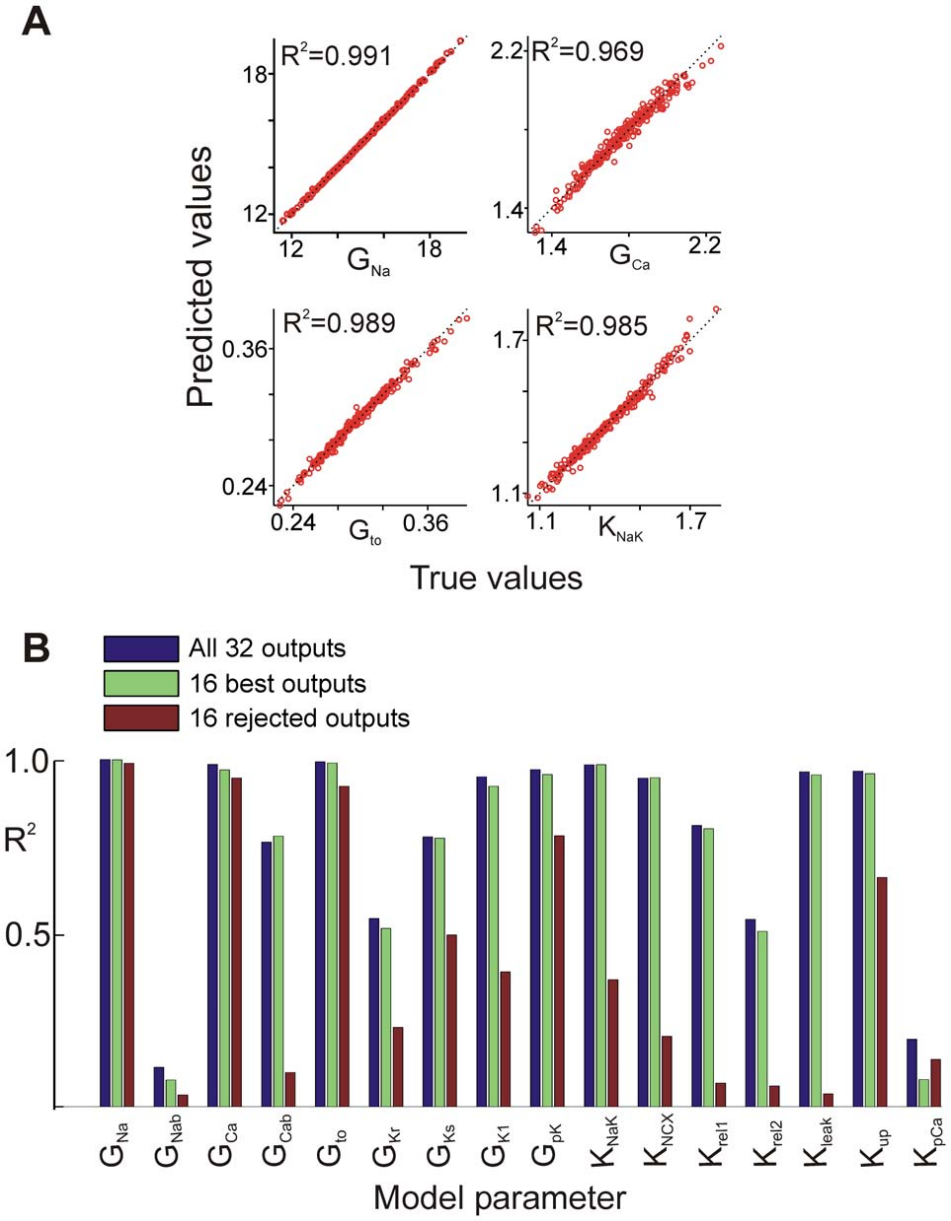
Predictions of the linear empirical model generated by reverse regression. (**A**) Scatter plots are displayed for four input conductances: G_Na_ (top left), G_Ca_ (top right), G_to_ (bottom left) and K_NCX_ (bottom right). Each plot shows the value actually used in the simulations (abscissa) versus the estimate generated by the regression model (ordinate). The regression was performed on a simulated data set containing 300 samples. (**B**) R^2^ values for each conductance in the TNNP model in the reverse regression. The three cases shown correspond to regression performed with: all 32 outputs (blue); the sixteen “best” outputs (green), and the 16 rejected outputs (red).

Figure 3A demonstrates the accuracy of the reverse regression method. For four chosen conductances, the scatter plots show the “actual” values, generated by randomizing the baseline parameters in the published TNNP model, versus the “predicted” values calculated with the regression model. The large R^2^ values (>0.9) indicate that the predictions of the regression method are quite accurate. Of the 16 conductances in the TNNP model, 12 could be predicted with R^2^>0.7. The four that were less well-predicted were the Na^+^ background conductance (G_Nab_), the rapid component of the K^+^ delayed rectifier conductance (G_Kr_), the sarcolemmal Ca^2+^ pump (K_pCa_) and the second SR Ca^2+^ release parameter (K_rel2_).

To verify that these encouraging results were not specific to the TNNP model, we performed similar analyses on additional models, the human ventricular myocyte model of Bernus et al. [17], and the “Phase 1” ventricular cell model of Luo and Rudy [18]. In either case (Figures S3 and S4, respectively), the reverse regression was highly predictive of most parameters, indicating that this approach is generally applicable. The outputs used for these analyses, listed in Text S1, differed somewhat from those used for the TNNP simulations because the Bernus et al. [17] and Phase 1 Luo and Rudy [18] models are relatively simple and do not consider intracellular Ca^2+^ handling in detail.

Figure 3B illustrates how the quantity and identity of the outputs in **Y** affected the accuracy of the predictions. Bar graphs show R^2^ values for prediction of each model parameter obtained by performing the reverse regression in three ways: 1) using all 32 outputs (blue), 2) matrix inversion (green), with the 16 best outputs as identified by the output elimination algorithm (see Methods), and 3) using only the 16 rejected outputs (red). The R^2^ values computed using the 16 best outputs were virtually identical to those obtained when all 32 outputs were used whereas R^2^ values for most conductances were substantially lower when only the 16 rejected outputs were included. These tests validate the algorithm which selected the outputs for matrix inversion. Moreover, since the 16 best outputs performed essentially as well as the full set of 32 outputs, this result implies that the model outputs were not fully linearly independent, and the 16 rejected outputs contained redundant information.

**Figure 4:**
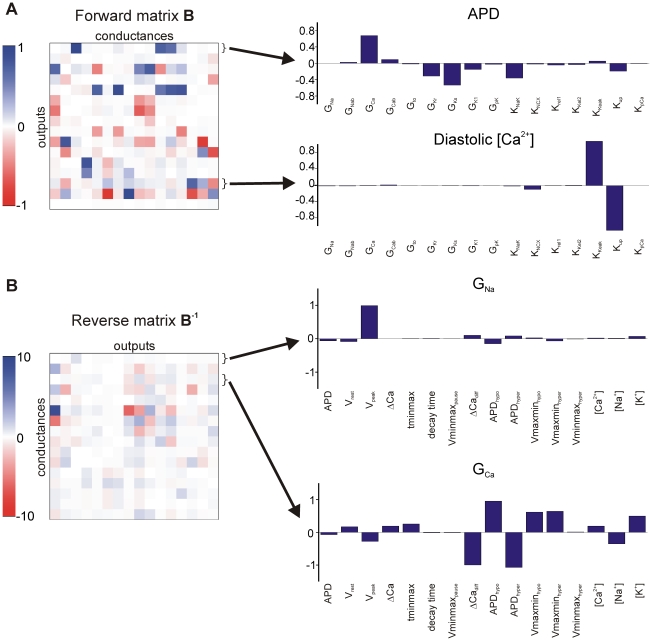
Parameter sensitivities for forward and reverse regression. Values in the forward regression matrix **B** and reverse regression matrix **B^−1^** are shown as “heat maps,” with white representing values near zeros, and blue and red indicating positive and negative values, respectively. (**A**) The forward regression matrix **B**, where each row represents the contributions of each of the conductances to a particular output. The bar graphs corresponding to two of these outputs (APD and diastolic [Ca^2+^]) are shown to the right. (**B**) The reverse regression matrix **B^−1^**, where each row represents the contributions of each of the outputs to a given conductance. The bar graphs corresponding to two of these conductances (G_Na_ and G_Ca_) are shown to the right.

Figure 4 displays, as heat maps, the coefficients for both the forward and reverse regression problems. The former indicate how model parameters influence outputs, whereas the latter specify how changes in model outputs restrict the parameters. Parameter sensitivities for selected outputs and conductances are shown as bar graphs to the right. As previously argued for the case of forward regression [16], these parameter sensitivities help to illustrate the relationships between parameters and outputs. For instance, forward regression coefficients indicate that diastolic [Ca^2+^] is determined primarily by a balance between SR Ca^2+^ uptake and SR Ca^2+^ leak, with other parameters making only minimal contributions. Conversely, for reverse regression, the maximal conductance of L-type Ca^2+^ current (G_Ca_) depends on many model outputs including action potential duration, Ca^2+^ transient amplitude, and, in particular, how these are altered with changes in extracellular potassium. This result underscores the centrality of intracellular Ca^2+^ regulation to many cellular processes.

**Figure 5:**
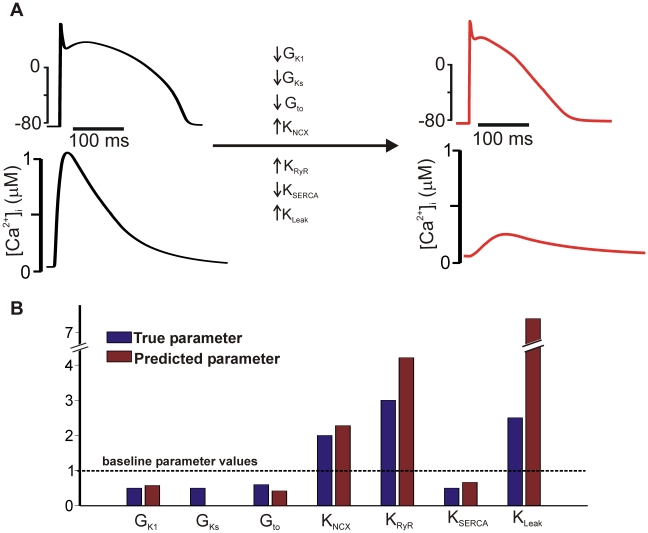
Application of reverse regression to constrain model parameters in heart failure. Simulations were performed with the Hund & Rudy model of the dog ventricular myocyte [19], with changes made to 7 model parameters to replicate changes occurring in heart failure, as previously simulated by Shannon *et al.* [20]. (**A**) The differences between normal and pathological states is shown by contrasting the action potential waveforms and Ca^2+^ transients. The action potential is triangular in shape in heart failure while the Ca^2+^ transient is dramatically reduced in the failing cell. The directional changes in the 7 altered parameters are also indicated. (**B**) The true values of the changed parameters are shown alongside the values predicted by reverse regression. Each is represented as a multiple of the baseline parameter value, where no change is indicated by the dashed line. Note the break in the y-axis, reflecting the fact that the reverse regression procedure overestimates the change in the parameter K_leak_. Similarly, the regression model overestimates the change in G_Ks_, as the height of this bar, 0.86% of the control value, is difficult to visualize on this scale.

The results shown in Figure 3 demonstrated that most of the model parameters used to generate the dataset could be reconstructed using the reverse regression procedure. To provide evidence that this procedure may be more broadly useful, we applied the method to a novel test case by performing simulations with the most recent version of the Hund & Rudy canine ventricular model [19]. Specifically, we considered changes in seven parameters corresponding to the condition of heart failure, as previously modeled by Shannon et al [20]. Figure 5A shows that implementing these parameter changes dramatically alters both AP shape and Ca^2+^ transient amplitude. After performing simulations under a range of conditions with both normal, healthy cells and pathological, failing cells (see Methods and Text S1), we asked how well the reverse regression matrix could calculate the parameter changes in the failing cells. We found that this method constrained 5 out of 7 parameters with excellent accuracy, while changes in two parameters (G_Ks_ and K_leak_) were overestimated somewhat by the regression algorithm. This novel test cases validates our approach and suggests that it may indeed prove a useful method for developing new models based on experimental measurements.

The second approach for constraining model parameters is based on Bayes's theorem. In statistics, this celebrated result describes the conditional probability of one event given another in terms of: 1) the conditional probability of the second event given the first, and 2) the marginal probabilities of the two events:
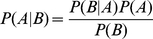
In this context, we consider event A that a model conductance lies within a given range, and event B that a model output is within a particular range. When many simulations are performed with randomly varying parameters, the probability P(A) is fixed by the user, while the probabilities P(B) and P(B|A) can be estimated from the results. This allows us to approximate P(A|B), which reflects how well a model parameter is constrained by a particular simulation result.

Since our hypothesis was that multiple outputs needed to be considered to constrain model parameters, we were interested in extensions of Bayes's theorem to more than two variables, e.g. P(A|B∩C), where B and C are events related to two model outputs. For instance, B and C could represent, respectively, that APD and Ca^2+^ transient amplitude are within particular ranges. If the conditional probability of the parameter increases as additional outputs are considered, this validates the thinking underlying the approach.

**Figure 6:**
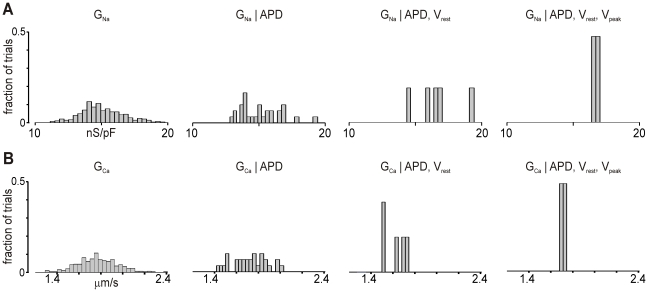
Illustration of Bayesian probability approach. (**A**) Distributions of G_Na_ with different constraints. From left to right, histograms show G_Na_ values in the complete data set; given that APD is in a particular range (from 295–298 ms, representing 10% of the samples); given that APD and V_rest_ (−84.96 to −85.02 mV) are in particular ranges; given that APD, V_rest_, and V_peak_ (37.05 to 37.81 mV) are in particular ranges. (**B**) Distributions of G_Ca_, given the same constraints as in (**A**).

The application of this strategy to our data set is illustrated in Figure 6. The two rows of histograms display distributions of G_Na_ and G_Ca_, which are typical of the 16 model parameters considered. The leftmost histogram in each row shows the distribution of conductance values in the entire population, and the remaining columns show conductance values for sub-populations that satisfy constraints on one or more model outputs. Successive columns from left to right show distributions with additional model outputs considered, as noted. In either case, the distributions become progressively narrower, and the conditional probability is unity once 3 outputs are considered.

This procedure also provides insights into which specific outputs provide the greatest information about particular model parameters. For instance, the distribution of G_Na_ given a certain range of APD appears similar to the overall distribution of G_Na_ because these two variables are not strongly correlated (i.e. P(B|A) ≈ P(B)). In contrast, inclusion of V_peak_, an output highly dependent on G_Na_, narrows the distribution significantly. In the case of G_Ca_, restricting APD to a particular range makes the distribution narrower, which is to be expected given the relatively strong correlation between the parameter and the output. Thus, an approach based on Bayes's theorem also supports the idea that model parameters can successfully be constrained if multiple model outputs are considered.

## Discussion

In this study we have presented two methods that can be used to constrain free parameters in complex mathematical models of biological systems. The utility of these methods was demonstrated through simulations with models of ventricular myocytes [15], [17]–[19], but with modifications the strategies could also be applied to other classes of models. For instance, these methods could be used to constrain parameters in models of the sinoatrial node [21], [22], but in this case more useful outputs would be metrics such as inter-beat interval, diastolic depolarization rate, and maximum diastolic potential [23]. Our results show that model parameters are difficult to specify uniquely using a limited number of model outputs as “targets,” but parameters can be constrained successfully if numerous model outputs are simultaneously considered [24]. The premise underlying this strategy is therefore similar to ideas advanced by Sethna and colleagues in discussions of model “sloppiness” [25], [26]. Even if individual parameters are largely unknown or cannot be measured with precision, predictive models can still be built if care is taken to match the model's output to diverse sets of experimental data.

The reverse regression method uses matrix multiplication to predict a set of parameters, in this case ionic current maximal conductances, that are most likely to recapitulate a given set of model outputs. In a recent paper [16], parameter randomization followed by regression was used to quantify parameter sensitivities in electrophysiological models. The method presented here is an extension of this: we added outputs so that the regression matrix **B** could be inverted. Each element of this inverted matrix, **B^−1^**, therefore indicates how much a physiological output contributes to the prediction of a particular input conductance (Figure 4). In experimental studies, metrics derived from data are frequently used as indirect semi-quantitative surrogates of ionic conductances. For instance, conventional wisdom holds that action potential upstroke velocity reflects the availability of Na^+^ current [27], and the prominence of the Phase 1 “notch” indicates the contribution of transient outward K^+^ current [28], [29]. Our reverse regression method is simply a mathematically more formal extension of this general strategy, whereby every output can conceivably influence the prediction of each model parameter.

When applied to the simulations with the TNNP model, reverse regression was able to generate accurate predictions of most conductances or rates of ion transport in the model (R^2^>0.7 for 12 of 16 parameters). Of the 4 parameters that were not predicted accurately, two, namely Na^+^ background conductance (G_Nab_) and the sarcolemmal Ca^2+^ ATPase (K_pCa_) are considered to be relatively unimportant for normal cellular physiology. The parameter K_rel2_ (c_rel_ in the original TNNP model), was also predicted poorly, most likely because it is partially redundant with the parameter K_rel1_ (a_rel_ in the original TNNP model), which was well constrained by the analysis. The surprise in our simulations was the poor prediction of the rapid component of the delayed rectifier current, G_Kr_, since this current contributes to AP repolarization [30], [31], and block of I_Kr_ is the primary cause of drug-induced long QT syndrome [32], [33]. It should be noted, however, that our prediction of the conductance corresponding to the slow delayed rectifier, G_Ks_, was accurate. This suggests that in the TNNP model, these conductances serve similar functions and perhaps compensate for each other.

A similar conclusion can be drawn from the simulations in which we used the reverse regression procedure to reconstruct the parameters corresponding to heart failure in the Hund & Rudy [19] model (Figure 5). Five out of the seven parameters altered in the heart failure cell were predicted accurately by the reverse regression procedure. The two that were not predicted accurately, K_leak_, and G_Ks_, have relatively minor effects in the Hund & Rudy model, although these are more important in some other models. Thus, these methods are not only useful for constraining parameters; they can provide novel insight into the relative importance of particular model parameters in determining physiological function.

Two important factors influencing the accuracy of the conductance predictions are the number and quality of the outputs. Mathematically, inversion of the regression matrix **B** requires that the columns be linearly independent, which in turn requires independence of the columns of **Y**, i.e. the outputs. In contrast, linear dependence would imply that the outputs contain redundant information. Since we did not know a priori which outputs would be informative and which would be partially redundant, we implemented an algorithm to remove outputs sequentially and find a set of 16 that yielded the best results. This resulted in the unexpected elimination of seemingly important outputs such as the maximal upstroke velocity, a metric closely related to Na^+^ conductance. However, it is important to note that this result does not argue against the usefulness of upstroke velocity as a metric, it merely indicates that the information contained in this output has already been captured by the 16 that were selected.

These considerations suggest a future application of these techniques, besides their obvious utility in the construction of new mathematical models. Since the regression analyses provide insight into which physiological measures are independent and which are partially redundant, these types of simulation studies can be used to prioritize experiments. Experimental studies consume the valuable resources of reagents, animals, and person-hours, and computational approaches that could reliably distinguish between more informative and less informative experiments would therefore be quite valuable. For example, the pacing cycle length at which a myocyte begins to exhibit APD alternans (BCL_alt_) is an important quantity related to the arrhythmogenic potential of the cardiac substrate [34], [35]. Determining this threshold, however, requires time-consuming experiments in which myocytes must be paced at many different rates. This output was rejected by our elimination algorithm, suggesting that, at least in the TNNP model, the information provided by this difficult experiment is not different from that contained in other, perhaps simpler, measurements. Our current work is focused on formalizing these ideas and developing methods to quantify the relative information content of different experimental measurements.

We should note that the outputs chosen for our analysis are physiologically meaningful metrics that are measured routinely in isolated cardiac myocytes. We purposely excluded measures that quantify how cellular behavior changes after application of a pharmacological agent. Since the explicit purpose of adding a drug is often to deduce the importance of the drug's primary target, we felt that including these metrics would, for an existing model, make the parameter constraint problem fairly trivial. In future studies, however, including these outputs will undoubtedly improve the predictive power of these methods. Similarly, the addition of more columns to the matrix **Y** corresponding to results from voltage-clamp experiments should also improve the accuracy of the method. These extensions will likely be necessary if maximal conductances are essentially unknown, or if ionic current kinetic parameters are also to be constrained.

In the field of cardiac electrophysiology, a few modeling studies have examined issues of parameter sensitivity [6],[16],[36],[37], parameter estimation [38], [39], and model identifiability [40]. For example, Fink and Noble recently assessed the adequacy of whole-cell voltage clamp records for uniquely determining parameters in models of ion channel gating [40]. These analyses suggested that optimized voltage clamp protocols might be more efficient for parameter identification than protocols currently used in experiments. More studies that address these sorts of issues have been performed in computational neuroscience. For instance, analogous to the results shown in Figure 1A, several studies have shown that different combinations of model conductances can produce seemingly identical behavior, either in isolated neurons [11], [13] or in models of small neuronal networks [14]. Olypher and Calabrese then generalized this result by demonstrating that, close to a particular location in parameter space, infinitely many parameter combinations can produce the same level of activity as the original location, and these authors derived 2×2 sensitivity matrices to demonstrate these compensatory changes [41]. Our reverse regression approach is essentially an extension of this idea to multiple dimensions, with the implicit assumption that considering additional linearly-independent model outputs will increase the likelihood of determining parameters uniquely.

Given that parameters in neuronal models cannot be uniquely specified using only a metric such as firing rate, a few studies have combined genetic algorithms with more sophisticated data-matching strategies such as phase-plane analysis [11] or multiple objective optimization [42]. Our methods offer both advantages and disadvantages compared with these alternative strategies. The primary advantage here is that reverse regression is simple and intuitive, and the outputs considered are well-defined metrics that are readily obtainable in the laboratory. We can therefore easily relate, in a way that other techniques do not allow, the observable characteristics of the cardiac myocyte to the membrane densities of the important ion channels. The main drawbacks of our approach are: 1) that we only perform a local search around the baseline model and 2) that we assume a linear relationship between changes in parameters and changes in outputs. While linear approximations to these input-output relationships have been shown to work well in cardiac models [16], particularly when conductances are expressed in log-transformed units, this assumption may not hold in all classes of models [43]. This limitation is evident in the simulations shown in Figure 6 in that: 1) two parameters were poorly predicted by the regression model; and 2) in these simulations, the parameter search was constrained to only seven possibilities rather than allowing any model parameter to contribute to the phenotype. Future studies will likely improve on these strategies and combine aspects of several approaches to refine methods for determining parameters in complex models of biological processes.

In summary, we have presented new methods for constraining free parameters in mathematical models, and demonstrated their utility through analyses of a common model of the ventricular myocyte. The approaches we describe have potentially broad implications. Analysis tools such as these can be used to obtain new insight into the relationships between model parameters, model outputs, and experimental data. The ideas offer hope that, even if some model parameters cannot be directly measured, a close comparison of data to model output can still discriminate between possibilities and produce a model with strong predictive power.

## Methods

This computational study aimed to extend the use of regression to develop methods for constraining free parameters in mathematical models. The ideas were tested through simulations using the TNNP model [15] of the human ventricular action potential (described in more detail in the Supporting Information). First, regression was used to derive a matrix **(B)** whose elements indicate how changes in input parameters, namely maximal ionic conductances, affect physiologically-meaningful model outputs. The regression matrix was then inverted, thereby deriving a new matrix **(B^−1^)** that specifies the ionic conductances required to produce a given set of model outputs.

In the first stage, the input matrix **X** was generated by randomly scaling 16 parameters in the TNNP model. A total of 300 random sets of parameters were generated such that **X** had dimensions 300×16. To compute the output matrix **Y**, several simulations were performed with each of the 300 models defined by a given parameter set. These simulations reflected standard electrophysiological tests such as the response of the myocyte to changes in pacing rate or extracellular potassium concentration. The calculation of some of these outputs is illustrated in Figure S1. The 32 outputs computed from these simulations, listed in Table 1, ranged from straightforward measures such as action potential duration (APD) and Ca^2+^ transient amplitude to more abstract metrics such as the minimum cycle length required to induce APD alternans [34].

The 16×32 matrix **B** relates the inputs to the outputs such that **Y** ^ = **XB** is a close approximation of the true output matrix **Y**. To allow for inputs and outputs expressed in different units to be compared, values in **X** and **Y** were converted into Z-scores – i.e. each column was mean-centered and normalized by its standard deviation. The results of the “forward” regression performed in the first stage are shown in Figure S2.

The second stage of the computational experiment aimed to determine if the input matrix **X** could be inferred, assuming the output matrix **Y** was known. Since **Y** ^ = **XB≈Y**, we reasoned that **YB^−1^** should be a close approximation of **X**, provided that **B** is an invertible matrix. We performed an iterative procedure to determine the 16 most appropriate outputs for this matrix inversion. First, with the full 300×32 matrix **Y**, “reverse regression” was performed to derive a matrix **B′** such that **YB′≈X**. We then removed each of the columns of **Y** and performed the reverse regression with the remaining 31 outputs. The output whose removal caused the smallest change in the prediction of **X** (quantified by R^2^) was deemed the least essential and was removed permanently. This procedure was repeated to reduce the number of outputs from 31 to 30, etc., until **Y** had dimensions 300×16.

A further set of simulations was performed with the 2008 version of the Hund and Rudy model of the canine action potential [19]. In these simulations, we sought to determine whether changes in model parameters in heart failure could be determined using the reverse regression procedure. We simulated the changes in parameters used by Shannon et al to simulate heart failure in their model of the rabbit action potential [20]. This involved alterations to seven model parameters: G_K1_, G_Ks_, G_to_, K_NCX_, K_RyR_, K_SERCA_, and K_leak_. Simulations were performed under three conditions: normal extracellular [K^+^]_o_ (5.4 mM), hypokalemia ([K^+^]_o_ = 3 mM) and hyperkalemia ([K^+^]_o_ = 8 mM). In these simulations, a total of 33 model outputs were calculated to constrain the parameters (see Text S1 for full list). Reverse regression was performed to map the 33 outputs from the simulated failing myocyte to the predicted 7 parameter changes.

In the second approach, based on Bayes's theorem, we were interested in estimating P(A|B) from P(B|A), P(A), and P(B). In this context, A is that a parameter is in a particular range, and B is that a model output is in a specified range. To estimate P(B|A) from the set of 300 simulation results, we sorted the values in each column of **X** and **Y**, then computed the percentile ranges. This allowed us to easily select, for instance, 10% of the values of a particular output centered around a given value. To generate histograms such as those shown in Figure 4, we first plotted the distribution of all the tested values of a given conductance. Then we selected the conductance values corresponding only to those trials for which APD fell within a particular range, and generated the histogram of this set. From this subset of conductances, we then selected the conductance values corresponding to those trials for which V_rest_ was in a certain range, etc. To allow for visual comparison, each histogram was normalized to the total number of values of the subset. To ensure that this procedure found a set of conductances that actually existed in the data set, we first identified the “best” trial for which the difference between **Y** and **Y** ^ was minimal. The output ranges used to select the subsets of conductances all represented deviations of ±5% around these values.

A bundle containing the Matlab™ code used to generate the results presented in the manuscript has been uploaded as Protocol S1 in the Supporting Information.

## Supporting Information

Figure S1 Examples of several of the outputs calculated from the simulations. (A) Shape parameters that are extracted from the AP waveform. These characterize the “spike and dome” morphology typical of epicardial myocytes. The loss of spike and dome indicates an abnormality possibly resulting from a pathological state. (B) Illustration of BCL_alt_ , the minimum basic cycle length required to induce APD alternans. When the cell is paced at BCL = 285, no alternans is observed. However, the characteristic alternating long-short pattern is seen at BCL = 280. (C) Illustration of the process used to determine the threshold stimulus current required to induce an action potential. Sub- and supra-threshold stimuli are applied iteratively until the correct magnitude of the threshold stimulus, in this case 15.9 pA/pF is determined. (D) Illustration of maximum slope of the APD restitution curve. Slopes>1 may indicate increased arrhythmia risk.
(0.63 MB EPS)

Figure S2 Bar graph showing the R^2^ values for each of the 32 outputs of the TNNP model predicted by the forward PLS regression. Most of these outputs (27 of 32) had R^2^ values>0.9. The outputs that could not be predicted well were among those that were rejected by the algorithm that narrowed the total number of outputs down to 16 for the matrix inversion.
(0.18 MB EPS)

Figure S3 Scatter plots showing the R^2^ values of the reverse regression predictions for 8 of the conductances in the Bernus [17] model. Prediction of mode of the conductances (6 of 8) was quite accurate (R^2^ values>0.7). The background Na^+^ conductance was poorly predicted; however, this conductance, however, plays only a minor role in the physiological behavior of the Bernus [17] model.
(1.25 MB EPS)

Figure S4 Scatter plots showing the R^2^ values for 6 of the conductances in the Luo-Rudy (LR1) model [18] predicted by the reverse PLS regression. All of these conductances had R^2^ values>0.65, and 5 out of 6 had R^2^>0.85.
(1.02 MB EPS)

Protocol S1 Bundle containing Matlab code used by the authors to generate the results presented in the manuscript. The file ‘READ ME’ within the bundle explains the function of each individual program.
(0.11 MB ZIP)

Text S1 Supplementary methods.
(0.09 MB DOC)
