# Supplementary material for: Regression Analysis for Constraining Free Parameters in Electrophysiological Models of Cardiac Cells
Source: PLoS Comput Biol. 2010 Sep 2;6(9):e1000914. doi: 10.1371/journal.pcbi.1000914 (PMC2932676; doi:10.1371/journal.pcbi.1000914)
Supplement: Text S1 — Supplementary methods. (0.09 MB DOC) [file pcbi.1000914.s006.doc]

**TEXT S1: Supplementary Methods**

**TNNP MODEL**

Simulations are performed with the human ventricular myocyte model published by ten Tusscher, Noble, Nobel and Panfilov (TNNP). The model is primarily based on data largely from human ventricular myocyte experiments, but in some cases ionic current formulations come from experiments in heterologous expression systems. The model simulates the major ionic currents including the fast Na^+^ current (I_Na_), the L-type Ca^2+^ current (I_Ca_), and various K^+^ currents including the fast transient outward current (I_to_), the inward rectifier current (I_K1_), and the rapid (I_Kr_) and slow (I_Ks_) delayed rectifier currents. The model also simulates triggering of sarcoplasmic reticulum Ca^2+^ release by I_Ca_ (Ca^2+^-induced Ca^2+^ release, CICR). It is able to reproduce heterogeneity in human epicardial, endocardial and M cell action potentials and demonstrate that observed discrepancies can be explained by variations in the transient outward and slow delayed rectifier currents. The epicardial myocyte was considered the baseline model in our simulations.

We attempt to constrain 16 model parameters, namely the maximal conductances of the major membrane currents and the maximal rates controlling pumps, transporters, and intracellular Ca^2+^ release channels. The specific parameters that were considered are listed in Table S1 along with their baseline values.

**Supplementary Table 1: Model parameters varied in TNNP s**imulations

| **Parameter** | **Definition** | **Baseline value** | **Notes** |
| --- | --- | --- | --- |
| G_Na_ | Maximal Na^+^ conductance | 14.838 nS/pF |  |
| G_Nab_ | Background Na^+^ conductance | 2.9 x 10^-4^nS/pF |  |
| G_CaL_ | Maximal L-type Ca^2+^ current permeability | 1.75 x 10^-4^cm3/μF. s |  |
| G_Cab_ | Background Ca^2+^ conductance | 5.92× 10^-4^ nS/pF |  |
| G_to_ | Maximal transient outward K^+^ conductance | 0.294 nS/pF |  |
| G_Kr_ | Rapid delayed rectifier K^+^ current scaling factor | 0.096 nS/pF | 1 |
| G_Ks_ | Maximal slow delayed rectifier K^+^ conductance | 0.245 nS/pF |  |
| G_K1_ | Maximal inward rectifier K^+^ conductance | 5.405 nS/pF |  |
| G_pK_ | Maximal plateau K^+^ conductance | 0.0146 nS/pF |  |
| K_NaK_ | Maximal Na^+^-K^+^ pump current | 1.362 pA/pF | 2 |
| K_NCX_ | Maximal Na^+^-Ca^2+^ exchange current | 1000 pA/pF | 2 |
| K_rel1_ | SR Ca^2+^ release scaling factor | 16.464 μM ms^-1^ | 3 |
| K_rel2_ | SR Ca^2+^ release scaling factor | 8.232 μM ms^-1^ | 3 |
| K_leak_ | Passive SR Ca^2+^ leak scaling factor | 8 x 10^-5^ ms^-1^ | 3 |
| K_up_ | Maximal rate of SR Ca^2+^ uptake (SERCA) | 0.425 µM/ms | 4 |
| K_pCa_ | Maximal sarcolemmal Ca^2+^ pump current | 0.825 pA/pF | 2 |

Notes

^1^The scaling factor for I_Kr_ is not formally a maximal conductance, since this is multiplied by √Ko/5.4 and can therefore be greater than this value. For a constant value of extracellular [K^+^], changing this factor serves to scale the current.

^2^Parameters controlling the magnitudes of Na^+^-K^+^ pump current, Na^+^-Ca^2+^ exchange current, and sarcolemmal Ca^2+^ pump current are all expressed as maximal current densities in units of pA/pF. The variable names used in the TNNP manuscript, are, respectively, *P*_NaK_, *k*_NaCa_, and (mistakenly) *G*_pCa_. To keep terminology consistent, we refer to these parameters here as K_NaK_, K_NCX_, and K_pCa_.

^3^The parameters controlling the maximal rates of SR Ca^2+^ release and passive SR Ca^2+^ leak, termed, respectively *a*_re1_, *c*_re1_ and *V*_leak_ in the TNNP study, are here, for consistency, called K_rel1_, K_rel2_ and K_leak_.

^4^In the TNNP model the parameter V_up_, in units of µM/ms, describes the maximal uptake through the SR Ca^2+^ pump. This is now termed K_up_ to be consistent with other parameters.

**DETAILS OF OUTPUT COMPUTATION**

The protocols implemented in order to measure each of the 32 outputs are described in brief below:

**Outputs derived from the AP waveform**

For the following outputs, 80 stimuli were applied at 1s intervals. Each electrical stimulus was a 2 ms current injection with magnitude 20 pA/pF. For each trial, outputs were extracted from the last AP of the series:

**Action potential duration (APD)**: Duration between the maximal rate of rise and the -60 mV crossing during the repolarization phase.

**Resting membrane potential (V_rest_)**: Membrane potential immediately prior to the electrical stimulus.

**Peak Voltage (V_peak_)**: This is the maximum voltage reached during phase 0 of the action potential.

**dV/dt_max_**: This is the maximum velocity achieved during the upstroke of the action potential.

The following shape parameters were extracted after computing the first and second derivatives of the action potential to determine maxima and minima as shown in figure S1 below:

**V_maxmin_**: This is the difference in voltage between the peak voltage of the action potential and the voltage at the lowest point in the notch.

**V_minmax_** : This is the difference in voltage between the highest point in the dome and the lowest point in the notch.

**t_minmax_**: This is the difference in time between the highest point in the dome and the lowest point in the notch.

**Outputs derived from the Ca transient**

**ΔCa:** This is the amplitude of the Ca^2+^ transient, calculated as the difference between the peak and the diastolic Ca^2+^ levels.

**Time to peak**: This is the time required for the Ca^2+^ transient to reach its maximum amplitude.

**Decay time**: This is the time required for the Ca^2+^ transient to decay to half of its peak value.

**Outputs derived after implementing pause protocol**

The following five outputs were measured after repeatedly stimulating the cell 40 times at 500 ms intervals before giving a long pause of 2500 ms and then stimulating the cell once more. The pause protocol is implemented as a disturbance in order to capture the tendency of the cell to display arrhythmogenic behavior. For example, the long QT syndrome is characterized by an increased risk of developing ventricular tachycardia such as Torsades de pointes. While the exact mechanism of TdP is not well understood, pause-induced early afterdepolarizations (EADs) are believed to be involved. Thus we measured several of our outputs after pacing the cell repeatedly at a clinically meaningful rate, followed by a long pause and then applying a single stimulus to study the effect of the long pause and thus quantify the predisposition of the cell to developing EADs. These five measurements were derived from the action potential fired after the long pause:

**APD_pause_**: This is the action potential duration after the pause.

**ΔCa_pause_**: This is the amplitude of the Ca^2+^ transient after the pause.

**Vmaxmin_pause**: This is the difference in voltage between the peak voltage of the action potential and the voltage at the lowest point in the notch measured after the pause.

**Vminmax_pause**: This is the difference in voltage between the highest point in the dome and the lowest point in the notch measured after the pause.

**tminmax_pause**: This is the difference in time between the highest point in the dome and the lowest point in the notch after the pause.

**Outputs derived from other stimulation protocols**

**BCL_alt_**: This is the basic cycle length required to induce APD alternans. It is known that cardiac myocytes, when paced at a sufficiently fast rate, exhibit an alternating long-short action potential pattern. The threshold rate is determined by pacing the cell at a slow rate at which alternans is not observed followed by a fast rate at which APD alternans is seen. The cell is then paced at a rate that is halfway between the two rates. If this rate too slow to induce alternans, then the point that is halfway between this rate and the previous fast rate is chosen for testing. Otherwise, if alternans is observed at that point, then the point halfway between the slow rate and this rate is tested. The stimulation interval is equal to the slow rate. Thus the minimum cycle length required to induce APD alternans is determined through this iterative averaging process. This method is illustrated in Figure S2 below.

**Frequency adaptation**: This is the change in APD that occurs in response to changing the frequency of pacing. For each trial, the cell is paced at a slow rate (BCL = 1000) and then at a fast rate (BCL = 400). The frequency adaptation is calculated as the difference in APD during the slow and the fast rates.

**I_thresh_**: This is the threshold stimulus required to fire an action potential. Like BCL_alt_, this value is also calculated through an averaging process. Sub and super-threshold stimuli are given to the cell and the average of the two values is used to test whether the cell is capable of firing an action potential under that stimulus. If so, the average of this value and the previous sub-threshold value is taken for testing. Otherwise, the average of this value with the previous super-threshold value is considered. This process is repeated until the correct threshold is determined, as illustrated in Fig. S3.

**Maxslope**: This is the maximum slope of the APD restitution curve. A restitution curve is generated by delivering premature stimuli at progressively longer intervals and plotting the action potential duration versus the prematurity of the stimulus. Twenty stimuli are delivered at a baseline rate of 600 ms and the action potential duration is calculated. Premature stimuli are given at diastolic intervals from 1 ms to 101 ms in 4 ms increments. The extracted output is the maximal slope of the resulting curve, as illustrated in Figure S4.

**Outputs derived from considering the difference between two AP waveforms**

The following two outputs were measured after repeatedly pacing the cell at regular intervals for each trial and then considering only the first and the last action potentials and corresponding Ca^2+^ transients from each trial:

**APD_diff_**: This is the difference in APD between the first and the last action potentials from each trial during pacing.

**ΔCa_diff_**: This is the difference in Ca transient amplitude between the first and the last Ca^2+^ transient in each trial.

**Outputs measured under hypokalemic conditions**

The following outputs were measured under conditions of hypokalemia (external [K^+^ ]= 2 mM).

**APD_hypo_**: This is the action potential duration under conditions of hypokalemia.

**V_maxmin hypo_**: This is the difference in voltage between the peak voltage of the action potential and the voltage at the lowest point in the notch measured under hypokalemic conditions.

**V_minmax hypo_**: This is the difference in voltage between the highest point in the dome and the lowest point in the notch measured under hypokalemic conditions.

**t_minmax hypo_**: This is the difference in time between the highest point in the dome and the lowest point in the notch measured under hypokalemic conditions.

**Outputs measured under hyperkalemic conditions**

The following outputs were measured under conditions of hyperkalemia (external [K^+^ ]= 8 mM).

**APD_hyper_**: This is the action potential duration under conditions of hyperkalemia.

**V_maxmin hyper_**: This is the difference in voltage between the peak voltage of the action potential and the voltage at the lowest point in the notch measured under hyperkalemic conditions.

**V_minmax hyper_**: This is the difference in voltage between the highest point in the dome and the lowest point in the notch measured under hyperkalemic conditions.

**t_minmax hyper_**: This is the difference in time between the highest point in the dome and the lowest point in the notch measured under hyperkalemic conditions.

**Outputs measured at steady state**

The following outputs were measured after a 1000 s pause:

**Ca^2+^**: This is the steady state intracellular Ca^2+^ concentration.

**K^+^**: This is the steady state intracellular K^+^ concentration.

**Na^+^**: This is the steady state intracellular Na^+^ concentration.

**FORWARD REGRESSION**

In order to generate the **X** matrix, the baseline parameters published in the TNNP model were scaled across 300 trials. Random scale factors for varying the conductances were chosen from a lognormal distribution with a median value of 1. The parameter σ, specifying the standard deviation of the distribution of log transformed variables, was set at 0.1. Once the 32 outputs were individually computed and their values were recorded in the **Y** matrix, the forward regression was performed. The matrix **B** of regression coefficients, computed when the regression was performed with only 16 of the most independent outputs, is shown in Fig. S5A. In this pseudo-color representation, white represents values close to zero, and blue and red indicate values above or below zero, respectively. The corresponding bar graphs for two of the outputs are also shown to illustrate how the various conductances contribute to these outputs.

Fig. S6 shows the predictive power (quantified as R^2^) of the regression model for all 32 outputs. As is evident, most of the outputs could be predicted with R^2^ > 0.9. The poorly predicted outputs were rejected by the algorithm that identified the 16 most appropriate outputs for inversion of **B**.

**REVERSE REGRESSION**

Fig S5B shows the inverse matrix **B^-1^** using the same pseudo-color representation as in Fig. 5A. In this case the values in **B^-1^** indicate how much each of the outputs contributes to constraining the model parameters. The corresponding bar graphs for G_Na_ and G_Kr_ are shown at the right. For the Na^+^ conductance, for instance, it is apparent that the largest contribution is from V_peak_. This is an expected result since I_Na_ is the largest current during the upstroke (Phase 0) of the action potential.

**REGRESSION APPLIED TO OTHER MODELS**

Before the regression method was attempted on the TNNP model, it was applied to two other models, namely, the Luo-Rudy (LR1) model and the Bernus model of the human ventricular action potential.

**BERNUS MODEL**

The forward regression was performed using the following 10 outputs:

APD

V_rest_

dV/dt_max_

V_peak_

V_maxmin_

V_minmax_

t_minmax_

BCL_alt_

I_thresh_

Maxslope

In the reverse regression the two outputs that were rejected by the algorithm described in the paper were t_minmax_ and maxslope. Thus the resulting **B^-1^** matrix had 8 rows and 8 columns, a necessary criterion for inversion. The scatter plots corresponding to the inversion are shown in Fig. S7.

**LUO-RUDY (LR1) MODEL**

The forward regression was performed using the following 10 outputs:

APD

V_rest_

dV/dt max

V_peak_

V_maxmin_

V_minmax_

t_minmax_

BCL_alt_

I_thresh_

Maxslope

Frequency

In the reverse regression the five outputs that were rejected by the algorithm described in the paper were BCL_alt_, V_peak_, t_minmax_ , maxslope and frequency. Thus the resulting **B^-1^** matrix had 6 rows and 6 columns, a necessary criterion for inversion. The scatter plots corresponding to the inversion are shown in Fig. S8.

**HUND AND RUDY MODEL**

In order to predict the 7 parameter changes that occurred to cause the heart failure phenotype in the hund model, we measured a total of 33 outputs under normal, hypokalemic and hyperkalemic conditions. These outputs were used in the reverse regression to specify the changes in the 7 input parameters. The 11 outputs measured under normal conditions were as follows:

**APD**

**V_rest_**

**V_peak_**

**Triangulation:** This measures the difference between APD_90_ and APD_30_. An action potential that is more triangular in shape is known to be indicative of heart failure.

**Calcium transient amplitude**

**dV/dt max**

**Diastolic [Ca^2+^] in the SR :** The spontaneous release of Ca2+ from the SR during diastole through mutant RyR2 channels increases the probability of arrhythmias. Thus this measure was included as an output.

**Time to peak**

**Decay time**

**V_maxmin_**

**V_minmax_**

In addition to making the above measurements during normal conditions, we also ran the same simulations under conditions of hypokalemia and hyperkalemia. This gave us a total of 33 outputs to use in the reverse regression to predict the 7 parameter changes.

**PROGRAM DETAILS**

All simulations were performed in MATLAB (The Mathworks, Inc., Natick, MA). Partial least squares regression was performed using the NIPALS algorithm implemented in the routines written by Dr. Herve Abdi of the University of Texas at Dallas.
