# Supplementary material for: Regression Analysis for Constraining Free Parameters in Electrophysiological Models of Cardiac Cells
Source: PLoS Comput Biol. 2010 Sep 2;6(9):e1000914. doi: 10.1371/journal.pcbi.1000914 (PMC2932676; doi:10.1371/journal.pcbi.1000914)
Supplement: Protocol S1 — Bundle containing Matlab code used by the authors to generate the results presented in the manuscript. The file ‘READ ME’ within the bundle explains the function of each individual program. (0.11 MB ZIP) [file pcbi.1000914.s005.zip › TNNP code/READ ME.doc]

**TNNP Model Code**

- The randomly generated parameter set is created by **generateparams**. The number of variations and the standard deviation (sigma) are specified in this program. This MUST be run first in order to derive the set of parameters **tnnp_fixed_params** that is read into each of the other simulations.
- The programs below should be run in the following order. They must all be run before the PLS regression can be performed with all 32 outputs:

(a) **tt_produce_aps:** This records the action potentials and calcium transients specified by each set of conductances generated in the previous step.
(b) **measure_ca_metrics:** This calculates the time to peak and decay time of the calcium transient recorded in the previous step.
(c) **tt_first_and_last_aps:** This records the first and the last action potentials and calcium transients for a given set of conductances.
(d) **APD_and_DCa_diff:** This calculates the difference in APD and amplitude between the first and the last APs and Ca^2+^ transients.
(e) **tnnp_derivs:** This measures the shape parameters that characterize the spike and dome morphology of epicardial cells.
(f) **tnnp_alternans:** This measures the minimum Basic Cycle Length (BCL) required to induce APD alternans.
(g) **tnnp_threshold:** This measures the current stimulus needed to fire an action potential.
(h) **tnnp_restitution:** This measures the maximum slope of the restitution curve.
(i) **tnnp_frequency:** This measures frequency adaptation.
(j) **pause_protocol:** This records the action potential after a long pause.
(k) **tnnp_derivs_pause:** This measures the shape parameters of the action potential waveform obtained after subjecting the cardiac myocyte to the pause protocol.
(l) **intracellular:** This measures the intracellular concentrations of Na^+^, K^+^ and Ca^2+^.
(m) **tt_produce_aps_hypokalemia:** This records the action potentials under conditions of hypokalemia.
(n) **tt_derivs_hypo:** This measures the shape parameters of the action potentials obtained under hypokalemic conditions.
(o) **tt_produce_aps_hyperkalemia:** This records the action potentials under conditions of hyperkalemia.
(p) **tt_derivs_hyper:** This measures the shape parameters of the action potentials obtained under hyperkalemic conditions.

- The program **performPLS_reverse_reject_extraoutputs** first performs the reverse regression with all 32 outputs and then sequentially rejects the 16 least independent ones until we are left with the top 16 outputs. It then does the reverse regression with only these 16 outputs.
- The program **performPLS_forward_extraoutputs** does the forward regression with all 32 outputs. This program must be run first before running **bayes_300**, which performs the Bayesian analysis.

**Hund Model Code**

- This can be found in a separate folder called **Hund** within the **TNNP** folder.
- As with the TNNP model, the first program that must be run to generate the parameter variations is **generateparams_hund**. This generates a data file called **hund_fixed_params**, which is then read into the next three simulations. The following simulations can be performed in any order:

(a) **hund08_isokalemia:** This program records the action potentials and calcium transients for each set of conductances generated in the previous step. The program then measures and records 11 different outputs extracted from these APs and Ca^2+^ transients.
(b) **hund08_hypokalemia:** This program records the action potentials and calcium transients for each set of conductances generated in the first step measured under hypokalemic conditions. The program then measures and records 11 different outputs extracted from these APs and Ca^2+^ transients.
(c) **hund08_hyperkalemia:** This program records the action potentials and calcium transients for each set of conductances generated in the first step measured under conditions of hyperkalemia. The program then measures and records 11 different outputs extracted from these APs and Ca^2+^ transients.
(d) **NZ_TRS_testseveral1:** This program calculates the 11 outputs for both normal and heart failure cells. It also allows us to vary the extent to which these 7 parameters are changed between the normal and diseased phenotypes.
(e) **NZ_TRS_testseveral2:** This program calculates the 11 outputs for both normal and heart failure cells measured under hypokalemic conditions. It also allows us to vary the extent to which these 7 parameters are changed between the normal and diseased phenotypes.
(f) **NZ_TRS_testseveral3:** This program calculates the 11 outputs for both normal and heart failure cells measured under hyperkalemic conditions. It also allows us to vary the extent to which these 7 parameters are changed between the normal and diseased phenotypes.
(g) **performPLS33_many:** This program performs the reverse regression with all 33 outputs and then uses the B_pls_^-1^ matrix derived in order to predict the parameters in the cardiac myocyte showing heart failure.
